# A Case of Reflex Irritation

**Published:** 1901-04-15

**Authors:** J. Taft

**Affiliations:** Cincinnati, Ohio


### A CASE OF REFLEX IRRITATION.

BY J. TAFT, M.D., D.D.S., CINCINNATI, OHIO.

Presented at Ohio State Dental Society.

Mrs. N., of comparatively good constitution and good health, whose teeth have been in the care of the writer for fifteen years or more, reports an affection which she has had during the last two weeks.

A whitlow or felon was formed on the index finger of the left hand, which, as such things usually do, gave very great pain. At the onset of this affection the teeth in both jaws became exceedingly sensitive, so that it gave pain to bring the teeth in contact. Pain was experienced even by pressure of the tongue upon the teeth. The gums about all the teeth became exceedingly sensitive and swollen. The patient could scarcely distinguish from which she suffered most—the whitlow or the diseased gums and sensitive teeth.

A strange feature about it was that there was a correspondence in the severity of pain between the two affections. When the pain was most intense in the finger the greatest pain was experienced in the gums. After the felon had formed, as she supposed, the finger was cut open, and very soon thereafter the pain ceased, as did also the pain in the teeth and gums.

As the finger returned to a healthy condition the gums did the same. The teeth were examined and not one was found to be in a condition that could in the slightest degree account for this affection of teeth and gums.

The teeth and gums altogether became affected in irritability, sensitiveness and swelling. These conditions subsided with the recovery of the felon. No treatment was given to the gums and teeth, and they are now in the usual good condition, perfectly natural in appearance, the teeth entirely free from any apparent abnormality.

Eight or ten of the teeth had at various times been filled with gold, but none within the last year and a half.

This is a brief outline made of the case as it presented itself, and it was an important and very strange one, that there should be that correspondence between affection of finger and tooth. You have all probably either heard of similar cases or at least read of such. Although these are two distinct parts of the human body, yet the tissues affected are somewhat analogous; it was the periosteum of finger and also of tooth which was affected. The periosteum about the root of a tooth is somewhat different from that of other bones; it is inclosed on both sides by bony structure, and it is also subjected to the great impacts of mastication; as pressure is made upon teeth this bears down on periosteum lining the sockets. The periosteum covering the bone of the fingers is also subjected to impacts, as there is pressure made in the seizing of objects and use of the fingers, but not so pronounced, however, as in the tooth. Whether this has anything to do with this correspondence in these conditions I can not say, but there is this analogy between them. It is a well understood fact that structures similar in character are likely to be effected wherever they exist in the body. If they are similar in their characteristics they will suffer under any general influence operating in the body. Another singular fact is that the whitlow is a local affection, but is not wholly independent of constitutional conditions which may co-exist. A condition must be favorable for it. Therefore where there is a similarity of structure and predisposition to affection, these like structures or tissues would be likely to suffer alike.

DISCUSSION.

Dr. C. R. Butler: An accident to my middle finger and bruise of the periosteum, which happened about five years ago, still gives trouble occasionally while operating. When exerting pressure on an instrument I find myself clinching my teeth in order to work with greater precision and more force. These influences are very peculiar and we all know how one part of a similar structure may be reflexly forced to work in accord with another, so why will not one portion take up the trouble of another in sympathy?

Dr. H. H. Harrison: Physiologists inform us that there is a particular area of the central nervous system which takes cognizance of tissue function in the several organs as well as the tissue of the body. If that be the case it should not be difficult for us to realize the corelations of all the structures of the body, and such a case as this would not be difficult to explain. It seems to me this was rather a coincidence than anything else. The felon on the finger caused the blood to be forced to that part at the same time it would be forced to the other parts, in abnormal rate and quantity. The effect would be the same, increased and diminished pain in each part from natural increased blood pressure. The effect of nerve impressions from the brain to the extremities is something which we have not understood or appreciated before, but the time is coming when we shall know very much more about the nervous system than we do to-day and can explain many things of which we are now ignorant.

